# The Physician Himself; 10th Edition

**Published:** 1892-09

**Authors:** 


BOOK ON THE PHYSICIAN HIMSELF, and Things that Concern His Reputation and Success. By. D. W. Cathell, M. D. New Tenth Edition (Author's Last Revision). Thoroughly revised, enlarged and rewritten. In one handsome Royal Octavo volume. 348 pages. Bound in Extra Cloth. Price, post-paid, $2.00, net. Philadelphia: The F. A. Davis Co., Publishers, 1231 Filbert street.

This book struck a popular chord, and has had a remarkable sale. There is that in it which appeals to the better sense of every physician, and no doubt it has done an immense deal of good in stimulating the profession to a more scrupulous observance of that *unwritten code* which should govern the conduct and shape the character of every one who wears the honored title "physician." It inculcates the highest principles; and even the highest and best of us may read it with benefit. The author has carefully revised his work and presents this the *tenth* edition much improved in the text and general get up.

